# Targeting microtubules sensitizes drug resistant lung cancer cells to lysosomal pathway inhibitors

**DOI:** 10.7150/thno.38729

**Published:** 2020-02-03

**Authors:** Tan-Min Chin, Gandhi T. K. Boopathy, Ellen P.S. Man, John G. Clohessy, Eva Csizmadia, Margaret P Quinlan, Thomas Putti, Seow-Ching Wan, Chen Xie, Azhar Ali, Fhu Chee Wai, Yan Shan Ong, Boon-Cher Goh, Jeff Settleman, Wanjin Hong, Elena Levantini, Daniel G. Tenen

**Affiliations:** 1 Cancer Science Institute, National University of Singapore, 14 Medical Drive, Singapore 117599.; 2 National University Health System, 5 Lower Kent Ridge Road, Singapore 119074.; 3 Parkway Cancer Centre, Gleneagles Hospital. 6A Napier Road, Singapore 258500.; 4 Institute of Molecular and Cell Biology, Agency for Science, Technology and Research (A*STAR), 61 Biopolis Drive, Proteos, Singapore 138673.; 5 Harvard Stem Cell Institute, Harvard Medical School, Boston, MA 02115.; 6 Massachusetts General Hospital Cancer Center, 149 13th Street, Charlestown, MA 02129.; 7 Institute of Biomedical Technologies, National Research Council (CNR), Pisa, Italy.; 8 Current address for Jeff Settleman: Pfizer, Inc. 10646 Science Center Drive, San Diego, CA 92121

**Keywords:** NSCLC, EGFR mutants, tyrosine kinase inhibitors, chloroquine, microtubule dysfunction

## Abstract

Oncogene-addicted cancers are predominantly driven by specific oncogenic pathways and display initial exquisite sensitivity to designer therapies, but eventually become refractory to treatments. Clear understanding of lung tumorigenic mechanisms is essential for improved therapies.

**Methods**: Lysosomes were analyzed in EGFR-WT and mutant cells and corresponding patient samples using immunofluorescence or immunohistochemistry and immunoblotting. Microtubule organization and dynamics were studied using immunofluorescence analyses. Also, we have validated our findings in a transgenic mouse model that contain EGFR-TKI resistant mutations.

**Results**: We herein describe a novel mechanism that a mutated kinase disrupts the microtubule organization and results in a defective endosomal/lysosomal pathway. This prevents the efficient degradation of phosphorylated proteins that become trapped within the endosomes and continue to signal, therefore amplifying downstream proliferative and survival pathways. Phenotypically, a distinctive subcellular appearance of LAMP1 secondary to microtubule dysfunction in cells expressing EGFR kinase mutants is seen, and this may have potential diagnostic applications for the detection of such mutants. We demonstrate that lysosomal-inhibitors re-sensitize resistant cells to EGFR tyrosine-kinase inhibitors (TKIs). Identifying the endosome-lysosome pathway and microtubule dysfunction as a mechanism of resistance allows to pharmacologically intervene on this pathway.

**Conclusions**: We find that the combination of microtubule stabilizing agent and lysosome inhibitor could reduce the tumor progression in EGFR TKI resistant mouse models of lung cancer.

## Introduction

Mutated tyrosine kinases in cancers resulting in “heavy dependence” to specific pathways have allowed the use of targeted therapy with an initial excellent therapeutic response 1. Endocytosis is a cellular process that selectively internalizes cell surface proteins by invagination of plasma membranes to endosomal vesicles and sorts for either degradation in lysosomes or recycling to the surface of cells 2. The ligand-bound/activated receptor-tyrosine kinases (RTK) are internalized by clathrin dependent and independent ways into early endosomes. From early endosomes, the receptors are transferred to late endosomes and eventually degraded in lysosomes or recycled back to the plasma membrane 3. This lysosomal degradation of RTKs attenuates the signaling triggered at the plasma membrane and reduces the number of available receptors at the surface for cellular homeostasis 3. It is known that cancers can have defective endocytosis and inefficient degradation of growth signals, resulting in excessively activated growth signals 4-7.

Here, we hypothesize that defective degradation of activated proliferative and survival signals in EGFR mutant lung cancers contributes to their dependence on the EGFR pathway. We describe in our study a defective endosome-lysosome degradation pathway resulting from a disorganized microtubule network in mutated Epidermal Growth Factor Receptor (EGFR) NSCLC cell lines and patient tumors. As a consequence of such disorganized microtubule networks, down-stream proliferative and survival signals are constitutively active. Our work provides evidence that the degradation pathway and microtubule network in these cancers are targets for novel therapeutic approaches. This concept complements the current understanding of how a hyperactive kinase can contribute to EGFR mutant lung cancers 8-10. Importantly, this observation may extend to other cancers with different mutated tyrosine kinases, representing a general advance in understanding how these cancers perpetuate.

## Materials and Methods

### Plasmid Constructs

cDNA for human EGFR wild type (NM_005228.3) and mutants (exon 19del and L858R) with C-terminal turbo-GFP were purchased from Gene Ethics. Also, to make the stable cells, we used EGFR-WT- with C-terminal GFP which was a gift from Alexander Sorkin (Addgene plasmid # 32751). EGFR-19-del (E746-A750) and L858R with C-terminal GFP were made from the EGFR-WT plasmid using QuikChange Site-Directed Mutagenesis kit (Agilent Technologies).

### Cell lines and stable cells

NCI-H1299 (EGFR WT), NCI-H1666 (EGFR WT), NCI-H460 (EGFR WT), NCI-H1650 and HCC4006 (EGFR exon 19 deletion mutants), NCI-H1782 (Her2 mutant) NSCLC cell lines, J82 (FGFR mutant) bladder cell line, HEK-293T, COS-7 and A431 epidermoid cell lines and primary immortalized human bronchial epithelial cells (BEAS-2B) were obtained from ATCC. The cells were maintained in RPMI or DMEM or Bronchial epithelial cell based medium containing 10% FBS. The PC9 cells expressing the EGFR exon 19 deletion mutation (E746-750) were provided by Kazuto Nishio (National Cancer Center Hospital, Tokyo) and maintained in RPMI containing 10% fetal bovine serum. MV4-11 and MOLM-14 (FLT3 mutant) leukemia cell lines were provided by Cheng Wee Joo (CSI Singapore) and maintained in RPMI containing 5% FBS. Stable cells were made using NCI-H1299 (EGFR WT) cells transfected with EGFR WT-GFP, EGFR 19del-GFP, or EGFR L858R-GFP by Amaxa Kit C, selected in G418 at 500 μg/ml and sorted for GFP. GFP positive clones were identified by Western blot.

### Pharmacologic agents

Erlotinib and Gefitnib were obtained from the NUH pharmacy. Chloroquine, Hydroxychloroquine, 17-AAG, Monensin, and Paclitaxel were purchased from Sigma Aldrich.

### Cell harvesting and protein analysis

Total cell lysates were prepared in modified RIPA lysis buffer (1% NP-40, 25mM Tris-HCl, pH 7.4, 150mM NaCl) with complete EDTA-free protease inhibitor and PhosSTOP Phosphatase Inhibitor Cocktail (Roche Diagnostics GmbH). Cell lysates were centrifuged 13,000 rpm for 20 min at 4 ºC and supernatants assessed by immunoblotting with the following antibodies: pEGFR (Tyr1068) from Biosource (#44-788G); EEA-1 (#2411), LAMP1 (#3243), Rab5 (#2143) Rab7 (#2094), Rab11 (#5589), cbl (#2179), pEGFR (Tyr1045) (#2237), pAKT (#9271), AKT (#9272), pERK (#9101), ERK (#9107), c-met(#3148), IGF-I Receptor (#3018), GAPDH (#2118), and Acetyl-α-Tubulin (#5335) from Cell Signaling; actin (sc-4778) and total EGFR (sc-03) from Santa Cruz; SNX1 (ab995) from Abcam. Anti-mouse and anti-rabbit secondary antibodies were from Cell Signaling Technology and Santa-cruz.

### Immunofluorescence, confocal microscopy, co-localization and live microscopy experiment

Cells were fixed with 3% paraformaldehyde or 100% Methanol for indirect immunofluorescence and then permeabilized in 0.1% Triton X-100. Cells were blocked in FDB buffer (5% FBS, 5% goat serum, 2% BSA in PBS, 1 mM MgCl2 and 1 mM CaCl2, respectively), and primary and secondary antibody incubations performed in FDB for 1 hour at room temperature. Cells were observed and images were taken using a confocal microscope (Nikon A1R and Olympus). Primary and secondary antibodies were used as 1:30 of EEA-1 (#2411; Cell Signaling), 1:50 of LAMP1 (ab25630; Abcam), 1:20 of Rab7 (#2094; Cell Signaling), 1:30 of Rab5 (#2143; Cell Signaling), 1:30 of Rab11 (#5589), 1:250 of GM130 (BD Biosciences) and 1:100 of β-tubulin (T4026; Sigma). 1:100 of γ-tubulin (T3559; Sigma), Alexa Fluor 488 (1:200), Alexa Fluor 555 (1:300) and Alexa Fluor 647 (1:50) were from Molecular Probes. For colocalization of EGF with lysosomes and early endosomes, fluorescence intensity ratios of EEA1 or LAMP1 with EGF-Alexa Fluor 488 (Invitrogen) for different time points were calculated using Fiji and signal intensities plotted against time for NCI-H1299 and PC9 cells.

### Immunohistochemistry

IRB approval was obtained under NHG DSRB B/07/463 and NHG DSRB B/08/196. Paraffin sections were deparaffinized, rehydrated to water, digested with DAKO Proteinase K and washed in TBS. Endogenous peroxidase activity was blocked with 3% hydrogen peroxide and incubated with EGFR (DAKO clone E30, Carpinteria, CA93013, USA) diluted 1:100 for 1 hour at RT. EGFR antibody was detected with the DAKO ChemMate Envision HRP kit, K5007. A semiquantitative method of scoring was used based on the extent and intensity of membrane staining: 0= negative staining; 1=<10% staining; 2=10 to 50% moderate to strong intensity; 3=>50% strong intensity. Scores 2 & 3 were considered positive.

For LAMP1 staining, sections were incubated with LAMP1 (Abcam, ab24170) diluted 1:200 for 2 hours at RT. The intensity of the staining along with its distribution (granular or diffuse) was noted. For the Ki67 staining performed on murine tumors, tissues were fixed in formalin and embedded in paraffin. Sections of 5 μm were obtained, air-dried, and baked for 30 min. Paraffin was removed with sequential washes in xylene, alcohol and water. Antigen retrieval was achieved by treating​with citrate buffer 10mM pH 6.0 for 1h. Sections were blocked with 7% horse serum (Normal Horse Serum Blocking Solution, Vector Laboratories #S-2000) for 30 minutes and incubated overnight at 4 °C with the primary antibodies (Ki67, Dako Cytomation, #M7240). Endogenous peroxidase activity was blocked by an incubation of 10 min in 0.03% hydrogen peroxide in PBS. Primary antibody staining was detected using biotin labeled Rabbit anti Rat secondary antibody (Vector Laboratories, #BA4001​) for 1h room temperature. The Avidin-Biotin complex-HRP (Vector Laboratories VECTASTAIN® Elite® ABC-HRP Kit (Peroxidase, Standard) #PK-6100) was added for half an hour and the signal visualized by using the DAB kit (Vector Laboratories DAB Peroxidase (HRP) Substrate Kit (with Nickel), 3,3'-diaminobenzidine SK-4100). Slides were counterstained with Gill II hematoxylin, after dehydration mount with cytoseal 60 (Electron Microscopy Sciences #18006).

### Cell viability assays

Approximately 7,500 NCI-H1299 cells were plated in 96-well plates and drugs added the following day, and media with drug changed every 2 days. Experiments were terminated when the control plate reached confluence. The remaining cells in each plate were stained with Giemsa, and the number of surviving cells assessed by TECAN (infinite M200).

For siRNA based knock-down analysis, around 3.5 x 10^4^ H1299 cells were plated and transfected with respective siRNAs using Lipofectamine RNAiMAX and analyzed after 48 hours of transfection. Erlotinib was added the next day, and siRNA was added again 24 hours later. Experiments were terminated 72 hours after the first addition of Erlotinib. Experiments were performed in triplicate and the number of surviving cells read by TECAN (infinite M200). The statistical significance of the difference in the number of surviving cells between control and knock-down was calculated with the two-tailed T-test using the Intercooled Stata 10.1 (StataCorp, Texas, USA) program. Transfection of sorting nexin 1 (SNX1) was performed using FuGENE6 and analyzed in the same manner.

### EGFR degradation assay

Cells were serum starved for 12 hours in OptiMEM (Invitrogen) and then treated with 100 ng/ml of EGF (Sigma). EGFR internalization and degradation were stopped by incubating the cells immediately in ice at the time points mentioned. The cell lysates for biochemical assays were prepared as described above. For immunocytochemistry assays, cells were serum starved, pre-treated with 100 ng/ml of EGF-Alexa Fluor 488 (Invitrogen) in ice to label the cell surface EGFR for one hour. Unbound EGF was removed by washing and labeled cells incubated at 37 °C for EGFR internalization. Cells were processed for immunofluorescence as described above.

### Mice and *in vivo* drug treatment

Genotyping of CCSP-rtTA and CCSP-rtTA-EGFR L858R-T790M alleles was carried out as described previously 11. Eight to 10 weeks old mice were fed with doxycycline to induce lung tumors. Lung tumor growth was detected and carefully followed by magnetic resonance imaging (MRI). After 5-6 weeks of induction, baseline MRI showed tumor growth in the lungs and at such time point, mice were randomized to vehicle (n=6), Paclitaxel (n=4), Gefitinib (n=4), Hydroxychloroquine (HCQ) (n=6), Paclitaxel and HCQ (n=6) or Gefitinib and HCQ (n=5) treatment. Mice were treated with Gefitinib (AstraZeneca, 50mg/kg in 0.5% HPMC and 0.2% Tween, daily oral gavage), Hydroxychloroquine (Sanofi-aventis, 180mg/kg in PBS, daily oral gavage), Paclitaxel (Selleckchem, 20 mg/kg in PBS, administered by IP injection three times per week i.e., Mon/Wed/Fri), Vehicle (0.5% HPMC and 0.2% Tween), or combination of Gefitinib plus Hydroxychloroquine, and Paclitaxel plus Hydroxychloroquine (at the above mentioned concentrations). MRI images were taken every 3 to 4 days to capture the effects of drug treatment on tumor size over 30 days. Processing and quantification techniques of tumor burden were based on manual segmentation/volume calculation of diffuse lung tumours as described previously 12. Changes in lung tumor volumes throughout the course of treatment were calculated as a percentage change in volume over tumor volume at day 1 of treatment, which was set at 100%. MRI images of mouse lungs were captured with a Bruker Biospec 94/20 9.4 Tesla scanner and the primary imaging sequence used was RARE (Rapid Acquisition with Refocused Echoes), with TR/TE=1200ms/17.5ms.

#### Study approval

All mice protocols were approved by the Institutional Animal Care and Use Committee (IACUC) at Beth Israel Deaconess Medical Center, Harvard Medical School, USA. This trial was approved by the National Healthcare Group of Singapore (NHG) DSRB/B/08/196 (Clinical trial NS01/03/08).

## Results

### EGFR mutants show a differential distribution of endosomal and lysosomal associated proteins

**Figure 1:**
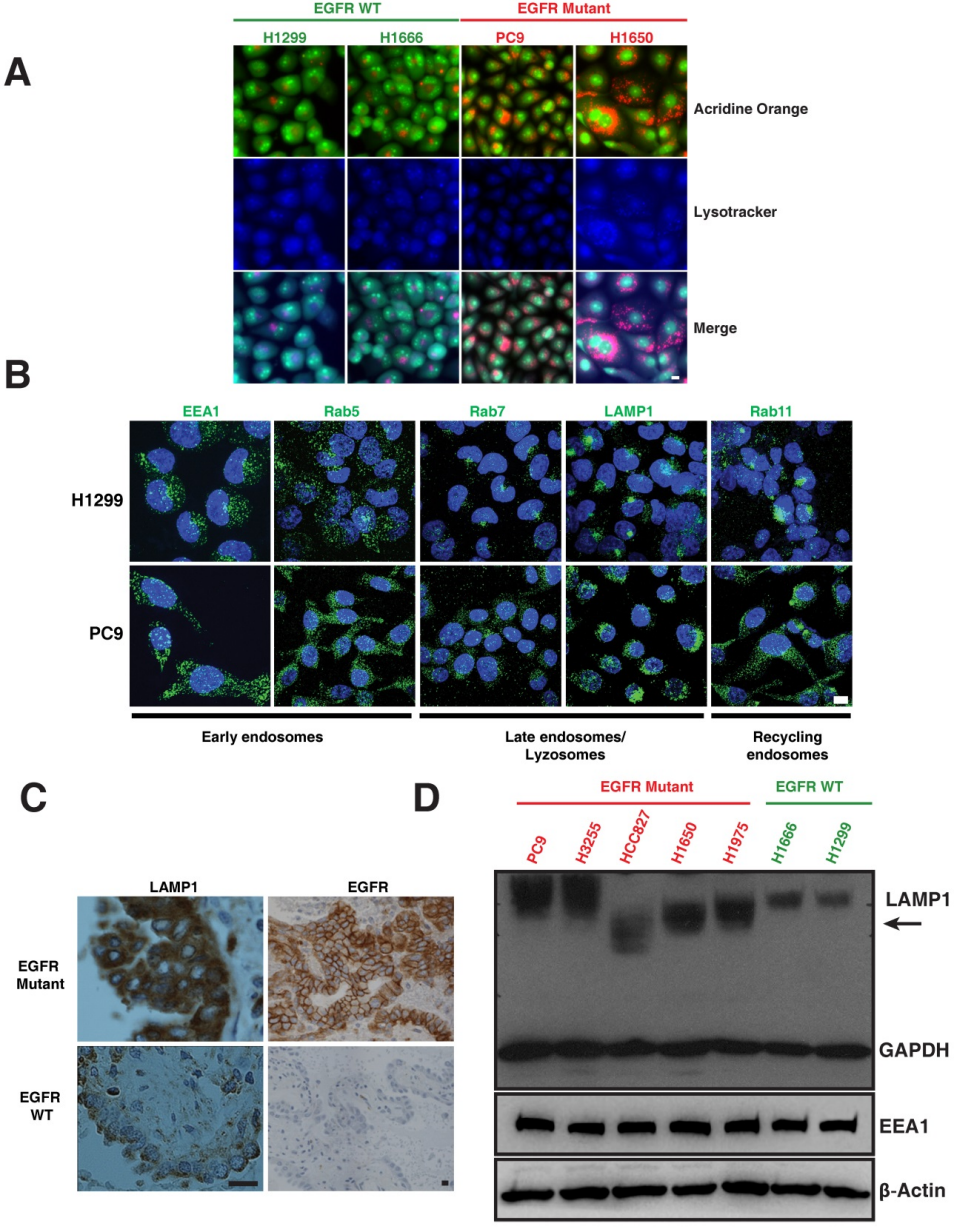
** Differential distribution of acidic and lysosomal organelles in EGFR wild-type versus mutant NSCLC cell lines and tumors. A**. Micrographs of EGFR wild-type (H1299 and H1666) and mutant (PC9 and H1650) NSCLC cells stained with acridine orange (green and orange) and lysotracker (blue). **B.** H1299 and PC9 cells were immunostained using antibodies specific for early endosomes (EEA1 and Rab5), late endosomes/lysosomes (Rab7 and LAMP1), and recycling endosomes (Rab11). The images represent maximum intensity projections using confocal microscopy. DAPI (blue) stains the nucleus. Scale bar: 10 μm. **C.** Immunohistochemical stains of LAMP1 and EGFR in primary human lung cancers. Staining demonstrates that the distribution of LAMP1 and EGFR (brown) in tumors closely resembles the distribution observed in NSCLC cell lines. Scale bar: 25 μm **D.** Western blot analysis of LAMP1 expression in NSCLC EGFR wild-type and mutant cell lines. Whole cell lysates were prepared, immunoblotted for early endosomes (EEA1), lysosomes (LAMP1) and loading controls (GAPDH and β-Actin).

The lysosomal pathway is crucial for degradation and thus downregulation of activated EGFR 13-15. We examined markers of the lysosomal pathway (endosomes-lysosomes) in both EGFR WT and EGFR mutant NSCLC cell lines. Endosomes and lysosomes have a low pH and are thus acidic organelles that can be identified by acridine orange staining. Early endosomes are distinguished by expression of Early Endosomal Antigen (EEA1) and Rab5; whereas late endosomes are identified by Rab7; lysosomes are identified by Lysosomal-Associated Membrane Protein (LAMP1), and recycling endosomes are identified by Rab11 staining. We observed a distinct difference in the distribution of acridine orange staining in mutant versus WT cells. To distinguish the nucleic acid binding capacity of the acridine orange staining, we have included lysotracker, a commonly used marker to label lysosomes. The merge panels indicating purple-shade clearly shows the overlap of lysotracker and acridine orange staining (Figure 1A). H1299 and H1666 cells (EGFR WT) showed a distinct, perinuclear localization of acridine orange (Figure 1A), as well as positivity for Rab7, Rab11 and LAMP1 (Figure 1B, top row) in the perinuclear localization of lysosomes in H1299 cells 16. In contrast, PC9 and H1650 cells (EGFR mutant) displayed a broadly diffuse, cytosolic distribution of acridine orange (Figure 1A). PC9 cells also demonstrated a punctate staining pattern of Rab7, Rab11, and LAMP1 throughout the cytosol (Figure 1B, bottom row). In both H1299 and PC9 cells, early endosomes are dispersed throughout the cytoplasm showing typical endosomal EEA1 and Rab5 staining with no detectable difference in localization between H1299 and PC9 cells. We also observed a similar differential expression of LAMP1 and EGFR in eight patient lung cancers that harbored mutant versus two EGFR WT (Figure 1C and Table S1). Western blot of LAMP1 in EGFR WT cells demonstrated a 100 kDa band, while mutant cells show a higher intensity with varying molecular weight bands (Figure 1D, arrow), suggesting a heterogeneous pool of lysosomes in mutants corresponding to LAMP1 staining seen in Figure 1B (lower panel).

### Mutations in the tyrosine kinase domain result in a defective endosome-lysosome maturation process

**Figure 2:**
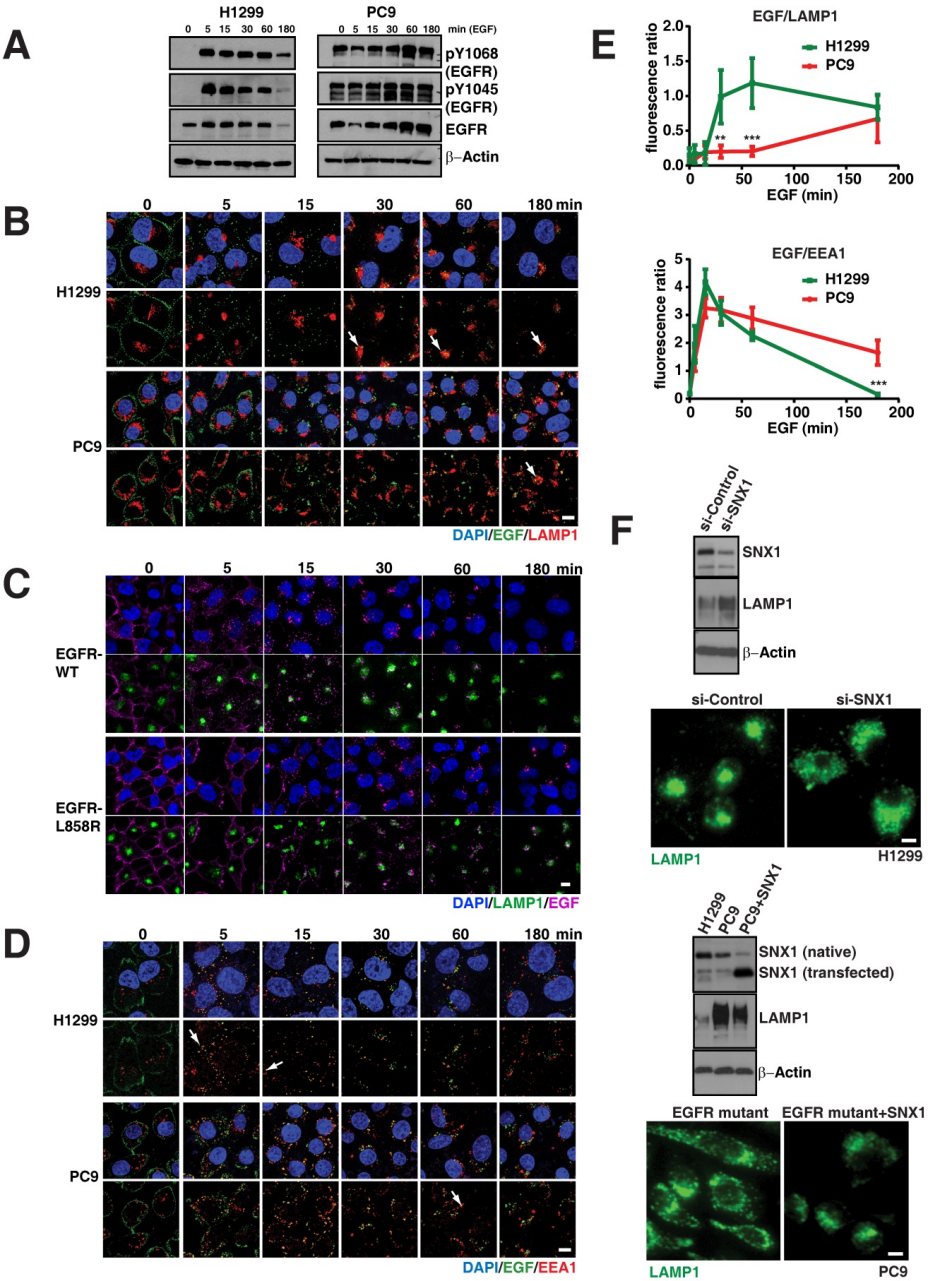
** EGFR mutants display delayed degradation of activated signals, resulting in increased and prolonged phosphorylated EGFR signaling. A.** EGFR degradation assay was performed on H1299 (EGFR-WT) and PC9 (EGFR-19del) lung cancer cells and cell lysates were immunoblotted for pY1068-EGFR, pY1045-EGFR, total EGFR and β-actin (as a loading control). EGFR degradation assay was performed and processed for immunofluorescence using the following cells and conditions: (**B**) LAMP1 antibody (red) for lysosomes and EGF (green) in H1299 and PC9 cells, (**C**) LAMP1 antibody (green) for lysosomes and EGF (purple) in and H1299-stable cells with EGFR-WT or EGFR-L858R (**D**) EEA1 antibody (red) for early endosomes and EGF (green) in H1299 and PC9 cells. The white arrows point colocalization of EGF with LAMP1 or EEA1. **E.** Quantitation of the fluorescent EGF intensity (green) overlapping with LAMP1 staining in panel B (top) or EEA1 staining in panel D (bottom) in H1299 (green line) and PC9 cells (red line) were plotted. Overlapping signal intensities were calculated using Image J. An average data of more than 100 cells from three independent experiments for each time point are plotted. Error bars represent standard deviation from the mean. ** and *** indicates *p*<0.001 and *p*<0.0001, respectively. **F.** Western blot and immunofluorescence studies on H1299 cell line and after knockdown of SNX1 (top blots and micrographs) and after transfection of SNX1 in PC9 cells (bottom blots and micrographs). Scale bar: 10 μm.

To assess if abnormalities in EGFR signaling are associated with altered endosomal and lysosomal pathways, we compared phosphorylated species of EGFR in WT and mutant cell lines. In particular, we observed that mutant PC9 cells display highly sustained levels of phosphorylated EGFR (both pY1068 and pY1045) upon EGF stimulation compared to EGFR WT cells (H1299), which demonstrate an efficient down-regulation of phosphorylated EGFR, (Figure 2A), similar to earlier reports in other cell lines 9, 10.

We next examined the endocytosis of EGFR in WT and mutant cells using Epidermal Growth Factor (EGF) conjugated with fluorescent dye-Alexa 488 and determined the accumulation of fluorescent intensity (as a readout for EGF) in lysosomes using LAMP1 as a marker at different time points. Figure 2B demonstrates fluorescent EGF efficiently shuttles to the lysosome starting approximately 30 minutes after EGF stimulation in EGFR WT and continued to accumulate until 180 minutes (Figure 2B, arrow). But, interestingly in EGFR mutants, EGF shuttling to the lysosome is severely delayed (starting around 60 min) and predominantly co-localized to lysosomes around 180 minutes after EGF stimulation (Figure 2B arrow). We quantified the overlap in fluorescent intensities of EGF and LAMP1 for both H1299 and PC9 cells at the time points in Figure 2B in three independent experiments. There is a significant difference in the colocalization of EGF with LAMP1 between H1299 and PC9 cells at 30 and 60 minutes (*p*-values 0.000197 and 0.000017, respectively) (Figure 2E; top).

To further confirm the differences in EGFR-WT versus the mutants in the endocytosis of EGF to the lysosomes is due the direct consequence of EGFR mutation, we developed EGFR WT and mutant (both 19del and L858R) stable cell lines using H1299 cells, which express low levels of EGFR WT (Figure S1A). Even after 4 hours of EGF activation, EGFR mutant stable cells display elevated levels of both phosphorylated and total EGFR levels when compared to EGFR-WT stable cells (Figure S1B). In EGFR WT cells, EGFR predominantly localized at the cell surface, while a large fraction of EGFR mutants remained in intracellular vesicles (Figure S1C). Using these stable cells, we performed the EGFR endocytosis assay in EGFR-WT and -L858R-mutant cells using EGF conjugated with alexa fluor 647. We found that most of the labelled EGF (magenta) effectively shuttles to the lysosome in around 30 minutes in EGFR WT cells, continued to accumulate in lysosomes (green), and at 180 minutes, the EGF intensity is highly reduced (Figure 2C; top two rows). But in EGFR-L858R cells, starting from 5 minutes, the endocytosis of EGF is delayed. At 30 minutes, most of the EGF is not localized to the lysosomes, and at 180 minutes, the EGF signal is stronger in EGFR-L858R cells than EGFR-WT cells (Figure 2C; bottom two rows), indicating that EGF is not degraded in EGFR-mutant cells. Taken together, data from cell lines and stable cells clearly demonstrate that EGF accumulation in lysosomes is strongly delayed in EGFR mutant cells.

As the early endosomes with EGFR mature to late endosomes and later to lysosomes, we further checked whether the EGFR mutant stayed longer in early endosomes. For this, we measured the colocalization of fluorescent EGF (as mentioned above) with EEA1, a marker for early endosomes, for three hours at multiple time points (Figure 2D). Overlap of EGF with early endosomes in H1299 cells starts as early as five minutes and peaks at 15 minutes, with negligible overlap at around 180 minutes (Figure 2D arrows). Similar to H1299 cells, PC9 cells colocalize early endosomes with EGF at five minutes with a peak at 15 minutes, but interestingly show a significant fraction of EGF remaining in early endosomes at 180 minutes when compared to H1299 cells (*p* = 4.64 x 10^-6^) (Figure 2E; bottom). Taken together, this data demonstrates that mutant EGFR remains longer in early endosomes, where signaling of activated EGFR can continue 16, 17.

In order to determine if the impaired trafficking of EGFR was a direct consequence of the kinase mutation or a result of “saturation” of the endosomal-lysosomal pathway 18 from a hyperactive kinase and excessive EGFR, we compared the transit of EGFR in A431 (a carcinoid cell line carrying EGFR WT and displaying high expression of EGFR as well as exquisite sensitivity to EGFR tyrosine kinase inhibitor (TKI)) to that in PC9 cells 19. Despite very high levels of EGFR, texas-red EGF demonstrates co-localization with LAMP1 in the typical lysosome location 60 minutes after EGF stimulation (Figure S2). In PC9, on the other hand, EGF does not show co-localization with lysosomes, rather the predominantly endosomal texas-red EGF (Figure S2 and Figure 2D, E). These experiments suggest that the impaired maturation from endosomes to lysosomes is a consequence of the mutation in the kinase domain and not simply the EGFR levels.

Previous reports have shown the importance that various motifs of the EGFR kinase domain have in engaging lysosomal targeting of messengers from the sorting nexin-1 (SNX1) and c-cbl family, with subsequent degradation of EGFR 20, 21. Indeed, siRNA against SNX1 (inducing a 60% decrease in endogenous expression) in the EGFR WT cell line (H1299) resulted in a change of total LAMP1 protein levels (Figure 2F, top blot) and distribution (Figure 2E, top micrograph), resembling the altered LAMP1 pattern seen in mutant EGFR (Figure 1D). Interestingly we observed that SNX1 was expressed at lower levels in PC9 cells. Transfection of SNX1 into these cells resulted in a change of the LAMP1 expression (Figure 2F, middle blot) as well as distribution to that of a perinuclear appearance (Figure 2F, bottom micrograph), resembling that of a WT cell line. These experiments suggest that the impaired maturation from endosomes to lysosomes in mutant EGFR may be overcome by overexpression of lysosomal targeting messengers.

### Defective endosome-lysosome maturation process in EGFR mutants is secondary to microtubule disorganization

**Figure 3:**
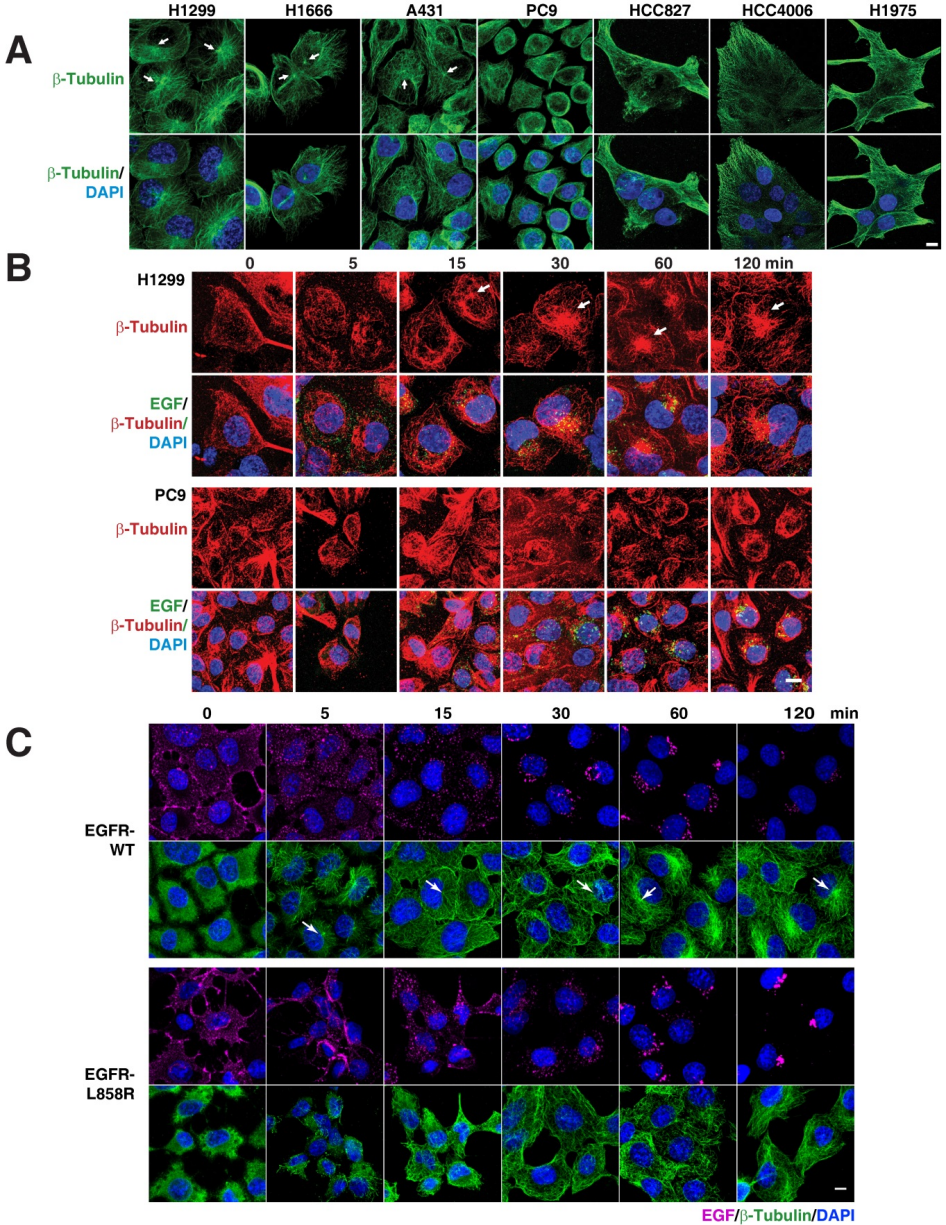
** EGFR mutants cause disruption of the microtubule network. A.** EGFR mutants have disorganized microtubule network. Immunofluorescence of β-Tubulin in EGFR wild type (NCI-H1299, NCI-H1666, and A431) and mutant cells (PC9, HCC827, HCC4006 and H1975). EGFR signaling stimulates microtubule organization. Pulse chasing of fluorescently labelled EGF was performed on the following conditions: (**B**) H1299 and PC9 cells with EGF (green) and subsequently processed for immunofluorescence using β-tubulin antibody (red). C, H1299 cells were stably transfected with EGFR-WT or EGFR-L858R were treated with EGF (purple) and subsequently processed for immunofluorescence using β-tubulin antibody (green). White arrows point to microtubule organizing center. DAPI stains nucleus. Scale bar: 10 μm.

As several membrane trafficking pathways rely on microtubules, we checked the distribution of microtubules in various EGFR WT (H1299, H1666, and A431) and mutant cells (PC9, HCC827, and HCC4006). Interestingly, we found that immunofluorescence of β-tubulin in EGFR WT and mutant cells revealed diverging patterns. While the microtubule network in EGFR WT cells was properly organized and concentrated with a distinct perinuclear microtubule organizing center (MTOC) (Figure 3A top panel; white arrows), in contrast in EGFR mutant cells, the microtubule network was not organized and did not originate from the MTOC (Figure 3A, top panel). We further checked for the microtubule organization in H1975, a TKI resistance cell line that harbor L858R-T790M mutation in EGFR. Similar to the above mentioned EGFR mutant cell lines, in H1975 cells, the microtubule network was not organized and did not originate from the MTOC (Figure 3A). Together, the data on microtubule organization and EGFR mutations show that multiple subtypes of EGFR mutations confer disorganized microtubules in NSCLC.

We also checked for the localization of lysosomes and early endosomes using immunofluorescence of LAMP1 and EEA1 in H1975 cells, respectively. Similar to our observation in EGFR mutant cells, LAMP1 staining showed a cytosolic distribution of lysosomes (Figure S3A). Also, the early endosomes are dispersed throughout the cytoplasm as observed in EGFR-WT and mutant cell lines (Figure S3B).

To determine if there is a relation between microtubule network organization and EGFR signaling, we used fluorescently labeled EGF and followed microtubule organization using β‑tubulin as a marker in H1299 and PC9 cells, as representative cells carrying WT and mutant EGFR, respectively. After 12-16 hours of serum starvation, β‑tubulin staining in H1299 cells showed a dispersed microtubule network in the cytoplasm (Figure 3B, upper panels). Fifteen minutes after EGF stimulation, H1299 cells showed a very well defined MTOC and radially arranged microtubules, with EGF locating around the MTOC region (Figure 3B, upper panel). In EGFR mutant PC9 cells, however, even after 120 minutes after EGF stimulation, microtubules demonstrated neither tubular structures nor concentrated at MTOC (Figure 3B, lower panel). These data suggest that mutant EGFR disrupts cellular microtubule organization.

An intact microtubule network plays a very important role in the organization and distribution of many cytoplasmic organelles, including the Golgi apparatus 22, 23. To further confirm the dysfunction of the microtubule network, we studied the distribution of the Golgi apparatus using an anti-GM130 antibody (GM130 is a cis-Golgi matrix protein) in multiple EGFR WT and mutant lung cancer cell lines. In EGFR WT cell lines, we found that the Golgi apparatus is stacked, continuous, and concentrated around the MTOC; while in the EGFR mutant cell lines, the Golgi apparatus is fragmented, scattered, and not concentrated at the perinuclear location of MTOC (Figure S4).

To study the disorganization of microtubules and the mislocalization of endocytic components as a direct consequence of EGFR mutation, we performed immunofluorescence of β-tubulin in H1299 cells (EGFR-WT and mutants). Quite interestingly, we found that both the exon 19 and L858R mutant cell lines demonstrated a defect in microtubule organization as assessed by β‑tubulin staining (Figure S5).

To further confirm the defective microtubule organization is due to the EGFR mutant signaling, we followed the microtubule network organization using β-tubulin staining upon the addition of fluorescently labeled EGF in a time resolved manner. Stable cells were serum starved overnight and then treated with fluorescent EGF, which was allowed to bind to cells for an hour in ice. The cells were then washed and incubated in a 37°C incubator. The cells were fixed at the time points mentioned and immunofluorescence of β-tubulin performed subsequently. In EGFR-WT cells, we found that microtubules start to organize from MTOC (white arrows in Figure 3) into a radial array to well defined microtubule filaments upon EGF treatment, starting from 5 minutes till the time point tested (120 minutes) (Figure 3C; top two rows). Quite contrastingly, in EGFR mutant cells, MTOC were not detected at any of the time point tested (Figure 3C; bottom two rows). The microtubule network of the EGFR mutant was more heavily disorganized at very early time points (5 and 15 min) than the EGFR-WT cells. These experiments strongly support the observation that mutant EGFR contributes to disrupting microtubule organization.

**Figure 4:**
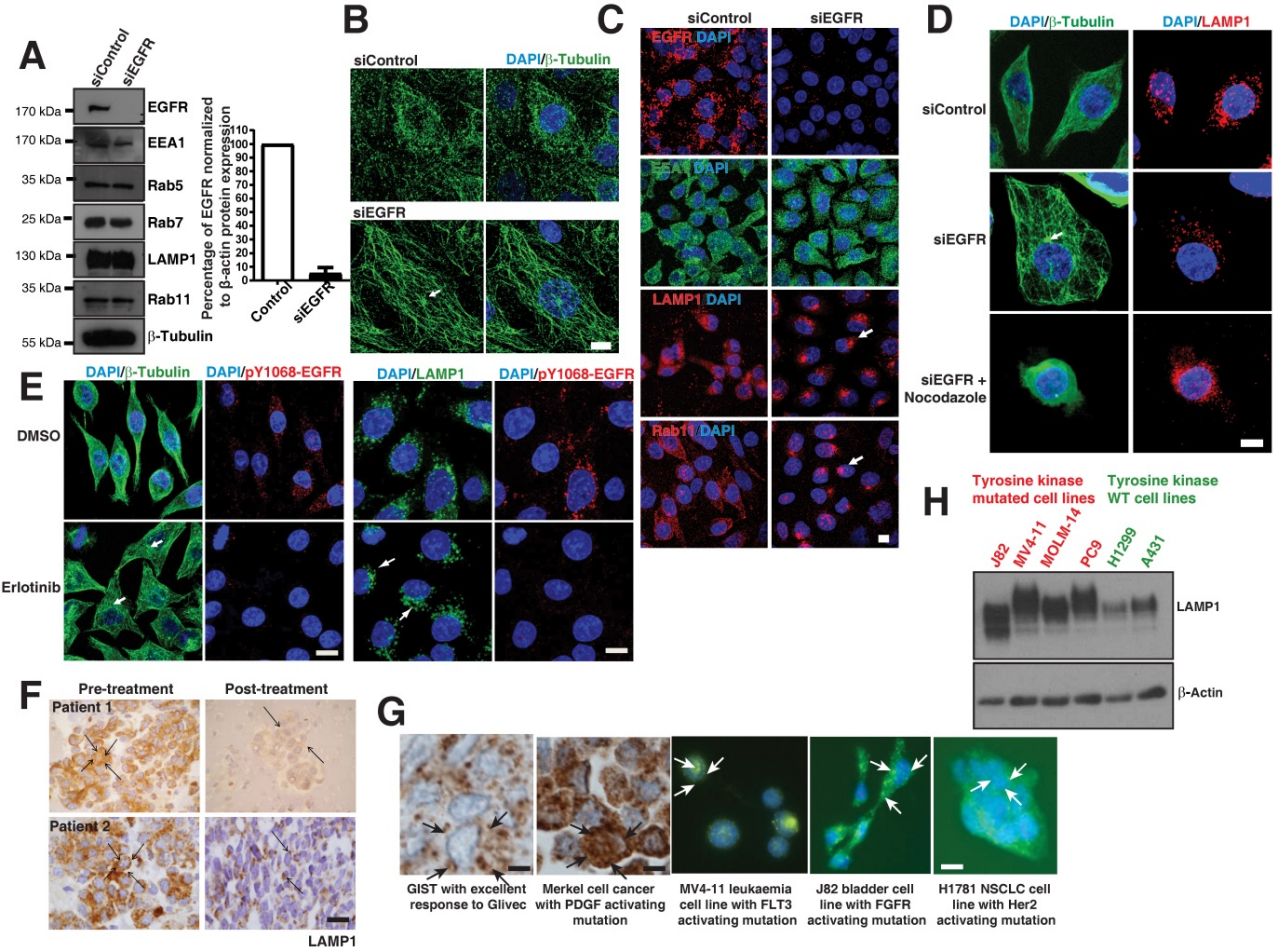
** Loss of EGFR mutant or its kinase activity causes microtubule re-organization. A.** Protein expression analysis of EGFR, endocytic proteins and β-Tubulin (for loading control) using immunoblot of H1650 cells with control siRNA and EGFR siRNA (left); Densitometry of percent of EGFR protein expressions were normalized to β-actin (right). **B.** Immunofluorescence of β-Tubulin (green) in control and EGFR mutant knockdown in H1650 cells. **C.** Immunofluorescence of EGFR and endocytic markers in control and EGFR knockdown in PC9 cells. White arrows point to perinuclear localization. **D.** Microtubule organization in EGFR mutant PC9 cells: Immunofluorescence staining of β-Tubulin and LAMP1 in control or EGFR knockdown or EGFR knockdown cells treated with 30 μM nocodazole. **E.** Erlotinib treatment of PC9 cells recovers microtubule organization and lysosomal localization: PC9 cells were treated with DMSO or 50 nM Erlotinib. Cells were fixed and analyzed for β-tubulin, pY1068-EGFR, and LAMP1 by immunofluorescence. White arrows point towards the microtubule organizing center (MTOC). Samples were recorded using confocal microscopy and represented in maximum intensity projections. DAPI stain indicates the nucleus (blue). Scale bar: 10 μm. **F.** Immunohistochemical studies on LAMP1 in EGFR mutant tumors pre- and post-EGFR TKI treatment, on the left and right, respectively. The LAMP1 distribution was studied in two paired patient tumor samples. Scale bar: 10 μm. **G.** The LAMP1 distribution studied both in patient tumors (using immunohistochemistry) and cell lines (immunofluorescence). Starting from the left panel, a patient with GIST and excellent response to Glivec; a patient with Merkel cell cancer and known PDGFR activating mutation in its tyrosine kinase domain; the leukemia cell line MV‑4‑11 with a FLT activating mutation; bladder cell line J82 with FGFR activating mutations; and NSCLC cell line NCI-H1781 with a Her2 mutation. The arrows in immunohistochemistry and immunofluorescence images demonstrate diffused LAMP1 distribution. Scale bar: 10 μm. **H.** Western blot analysis comparing the expression of LAMP1 in multiple cell lines with receptor tyrosine kinase mutations (J82, MV4-11, MOLM-14, and PC9 cells) and WT receptor tyrosine kinases (H1299 and A431 cells). β‑Actin was the loading control.

To validate our hypothesis that such disorganization is a direct consequence of the mutant EGFR, we knocked down EGFR in EGFR mutant cells (PC9 or H1650) using a pool of EGFR specific siRNAs (Figure 4A; left), showing a 95% efficiency of knockdown (Figure 4A; right). We found that the knockdown of EGFR does not cause major alteration to the levels of endosomal compartments such as early endosomes (EEA1 and Rab5), late endosomes and lysosomes (Rab7 and LAMP1), and recycling endosomes (Rab11) (Figure 4A). Strikingly, we found that knocking down EGFR in mutant cells results in a very well organized microtubule network with visible MTOC in H1650 cells (Figure 4B). This is quite interesting, as the disorganization of microtubule due to EGFR active mutants is not reported earlier.

We next checked if knocking down of the EGFR mutant would cause perinuclear localization of lysosomes and recycling endosomes in EGFR mutant cells. Interestingly, LAMP1 (lysosomes) and Rab11 (recycling endosomes) on EGFR mutant knockdown cells localized to the perinuclear region similar to EGFR WT cells (Figure 4C). However, the localization of the early endosomes (EEA1) did not change with the presence or absence of an EGFR mutant (Figure 4C middle panel).

To validate that the perinuclear localization of lysosomes upon loss of EGFR mutant is due to an organized microtubule network, we performed knockdown of EGFR in PC9 cells. The knockdown of the EGFR mutant rescued the organization of microtubule and the lysosomes to the perinuclear space (Figure 4D, middle panel). Also, we treated EGFR knockdown cells with the microtubule depolymerizing agent nocodazole (Figure 4D; bottom panel). We observed that the lysosomal vesicles were mislocalized and distributed throughout the cytoplasm in nocodazole treated EGFR knockdown cells (Figure 4D, bottom panel), clearly suggesting that the rescued perinuclear localization of lysosomes upon knockdown of mutant EGFR is due to organized microtubules.

To confirm that the constantly active kinase of the EGFR mutant is the cause for microtubule defect, we inhibited the kinase activity by using Erlotinib. Block of the kinase activity in PC9 cells was assessed by immunofluorescence staining of anti-pY1068-EGFR (Figure 4E, red staining) and resulted in organized microtubule structures, as assessed by β-Tubulin staining (Figure 4E, green staining in left panels). We also checked lysosomal localization after TKI treatment, and LAMP1 immunofluorescence showed perinuclear localization of lysosomes (Figure 4E, green staining in right panels), as observed in EGFR knockdown in these cells (Figure 4C, middle panels).

Similarly, we checked for LAMP1 localization in NSCLC patients carrying mutant EGFR tumors before and after Gefitnib treatment. In eight patients, we observed a diffusely cytosolic distribution of LAMP1 and a good response to EGFR TKIs. Interestingly, two patients who initially responded to TKI demonstrated a change in the LAMP1 distribution at the time of progression of the disease, when the tumor stopped responding to EGFR TKI (Figure 4F). Taken together, these experiments further demonstrate that a constantly active mutant EGFR induces microtubule disorganization in cancer cells and causes LAMP1 mislocalization.

Delayed degradation of phosphorylated signals may occur in c-kit 24 and PDGF 25 mutated tumors. We looked to see if the diffuse cytoplasmic distribution of LAMP1 may also apply to these other cancer types. We noted a diffuse distribution of LAMP1 in patient tumor samples with Gastrointestinal stromal tumor (GIST) and Merkel cell cancers with PDGF mutations using immunohistochemistry of LAMP1 (Figure 4G, left panels). Also, immunofluorescence of LAMP1 similarly a diffused cellular distribution of lysosomes in cell lines from leukemia (MV‑4‑11), bladder (J82), and NSCLC (H1781) cancers with FLT3, FGFR, and Her2 tyrosine kinase mutations, respectively (Figure 4G, middle and right panels). Interestingly, similar to that seen in EGFR mutant cells, LAMP1 appeared as a diffuse band on western blot analysis in receptor tyrosine kinase mutated cells (J82, MV4-11, MOLM-14, and PC9 cells) compared to WT cells (H1299 and A431 cells) corresponding to a heterogeneous pool of lysosomes (Figure 4H). These results suggest that across multiple cancer types, kinase mutations affect the degradation of activated signals, contributing to oncogenesis.

### Targeting the lysosomal pathway sensitizes resistant tumors (both primary and acquired resistance) to EGFR TKIs

**Figure 5:**
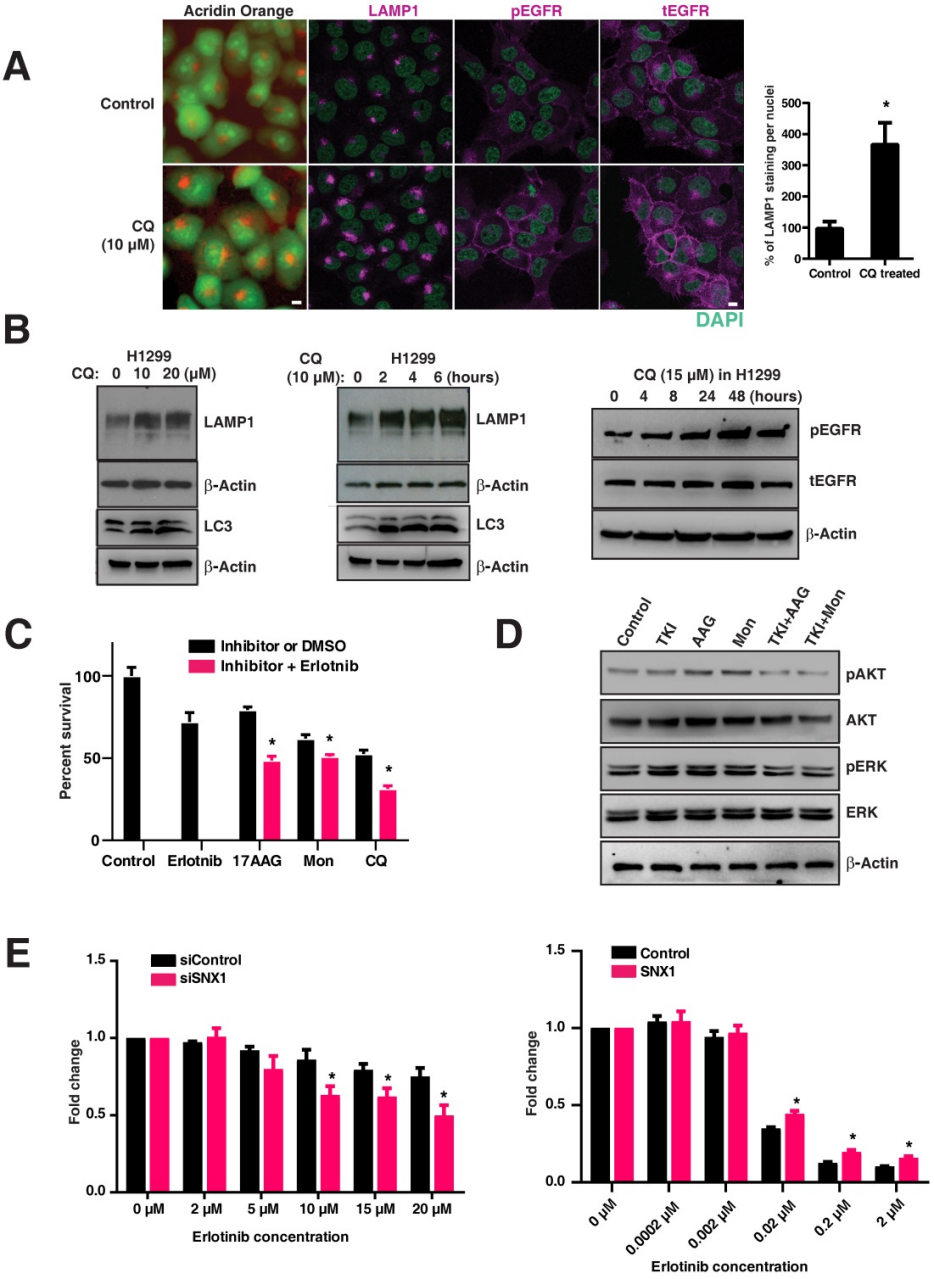
** Targeting the lysosomal pathway may sensitize resistant tumors to EGFR tyrosine kinase inhibitors. A.** Acridine orange staining and immunofluorescence analysis of LAMP1, pY1068-EGFR, and total EGFR in H1299 cells (EGFR WT cell line resistant to TKIs) treated with chloroquine or vehicle for four hours. DAPI stains thenucleus (green). The percentage of LAMP1 staining per nucleus in CQ treated cells with respect to the control cells is shown in the bar graph. **B.** Expression of LAMP1 and LC3 in chloroquine-treated H1299. H1299 was treated with increasing concentrations of chloroquine for four hours (10μm and 20μm) (left panels), and for an increasing duration with 10μm of chloroquine (middle panels). The right panel shows corresponding to an increased total LAMP1 and an increased amount of phosphorylated and total EGFR in cells treated with 15μM chloroquine. **C.** The cell viability assay of inhibitors when used alone and when used in combination with erlotinib are shown. The inhibitors used are erlotinib (2μM), 17-AAG (0.025μM), Monensin (0.02μM), and Chloroquine (5μM). The difference in cell viability of each inhibitor used in-combination with erlotinib (2μM) is shown next to the bar chart. Results shown here are representative of three independent experiments, and error bars represent standard deviations from the mean. **D.** Western blot of the downstream signaling events after drug treatment with EGFR TKI (Erlotinib), 17-AAG (AAG), or Monensin (Mon) on H1299 cells. Phospho-AKT (pAKT), total AKT, phospho-ERK (pERK), and total ERK were assessed at 8 hours after H1299 cells were treated with either 2μM of Erlotinib (TKI), 17-AAG (0.005μM), or Monensin (0.005μM), or a combination of Erlotinib and 17-AAG (TKI+AAG) or Erlotinib and monensin (TKI+Mon), respectively. **E.** Cell viability analysis of erlotinib treatment on EGFR wild type cells (H1299) with either control siRNA or SNX1 siRNA (left panel), and PC9 cells transfected with either control or SNX1 plasmids (right panel). SNX1 knockdown cells were treated with increasing concentrations of erlotinib from 0 to 20 μM for 72 hours, and the fold change of surviving cells with respect to untreated cells in each set of experiments is shown. Each data point represents an average of three independent sets of experiments, and errors bars show the standard deviation from the mean. The respective two-tailed p-values at each concentration of erlotinib (2 μM, 5 μM, 10 μM, 15 μM, and 20 μM) are 0.333464, 0.078570, 0.009807, 0.010858, and 0.007565, respectively. PC9 cells were transfected with either control or SNX1 for 48 hours, and their sensitivity to erlotinib determined. Transfected cells were treated with increasing concentrations of erlotinib from 0 to 2 μM for 72 hours, and the percentage of surviving cells with respect to untreated cells in each set of experiments is shown in the chart. Each data point represents an average of three independent sets of identical experiments, and errors bars show the standard deviation from the mean. The respective two-tailed p-values at each concentration of erlotinib (0.0002 μM, 0.002 μM, 0.02 μM, 0.2 μM, and 2 μM) are 0.949983, 0.480825, 0.001439, 0.001235, and 0.001518, respectively). * indicates *p*-value <0.05.

We postulated that inhibition of EGFR degradation using chloroquine in EGFR WT tumors (resistant to EGFR TKIs) would render them more dependent on the EGFR pathway and thus more sensitive to EGFR blockade. To test this, we treated the H1299 cell line (resistant to EGFR TKIs) with an inhibitor of the lysosomal pathway (chloroquine) that impairs the maturation of endosomes to lysosomes. Interestingly, chloroquine treatment changed the LAMP1 distribution from that of lysosomal to early and late endosomal (Figure 5A), similar to that observed in mutant lines (Figure 1B). A significant and dose dependent increase in total LAMP1 levels was observed in chloroquine-treated H1299 cells (p=0.00248) (Figure 5A graph, Figure 5B, left panel), as well as a time-dependent increase (Figure 5B, middle panel). These increase in LAMP1 levels were corroborated with the conversion of the autophagic marker LC3-I (top band) to LC3-II (bottom band) in H1299 cells (Figure 5B, middle panel: bottom bands). Correspondingly, an increased amount of pEGFR (pY1068-EGFR) was observed in chloroquine-treated cells, suggesting that delayed maturation of endosomes-to-lysosomes resulted in a delayed degradation of pEGFR (Figure 5B, right panel). Also in H1299 cells, the total EGFR levels have increased upon chloroquine treatment as observed in Figure 5A, suggesting a hampered EGFR degradation. Importantly, when lysosomal inhibitors (chloroquine or monensin) and heat shock protein inhibitors (17-AAG; affecting stability and folding/unfolding of proteins) are used in combination with EGFR TKIs, the H1299 cells, originally resistant to EGFR TKIs, are rendered significantly sensitive to cell death (p-values: 17-AAG vs 17-AAG with Erlotinib; monensin vs monensin with Erlotinib; and chloroquine vs chloroquine with Erlotinib are 0.0059, 0.0329, and 0.0103, respectively, with a strong downregulation of phospho-ERK and phospho-AKT (Figure 5C and 5D).

To determine if altering the expression of SNX1 (previously shown to change LAMP1 distribution as shown in Figure 2E) would change the sensitivity to EGFR TKIs, we knocked down SNX1 in H1299 cells followed by treatment with Erlotinib. We observed a decrease in cell viability of H1299 cells upon knockdown of SNX1 (Figure 5E left), consistent with our observation that disruption of the lysosomal pathway results in increased sensitivity to EGFR TKIs. Conversely, transfecting SNX1 rendered PC9 (usually sensitive to erlotinib) mildly resistant (Figure 5E, right) and correlated with the change in LAMP1 distribution from cytosolic to focal (Figure 2E), demonstrating that partial correction of the defective endosomal-lysosomal trafficking pathway can introduce resistance to EGFR tyrosine kinase inhibitors. These data show that lysosomal inhibitors along with EGFR TKIs sensitize cells originally resistant to EGFR TKIs (Figure 5C).

### Targeting the lysosomes or microtubules sensitize tumor cells with acquired resistance to EGFR TKIs in cell culture and *in vivo*

**Figure 6:**
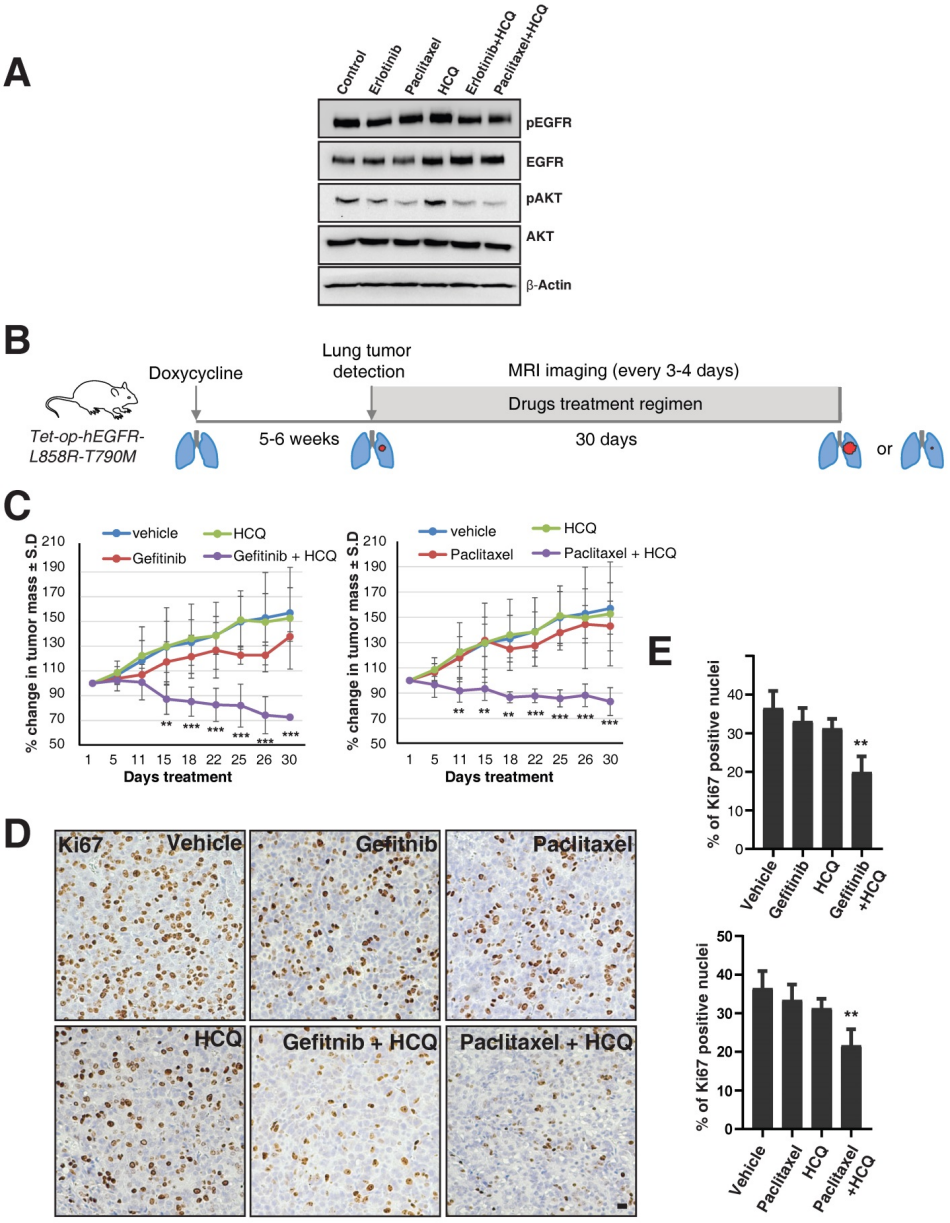
** Targeting the lysosomes or microtubules sensitizes mice with resistance to EGFR TKIs. A.** EGFR TKI resistant H1975 cells were treated with erlotinib, paclitaxel, hydroxychloroquine (HCQ), a combination of erlotinib with HCQ, and a combination of paclitaxel with hydroxychloroquine (HCQ). Cell lysates were harvested and immunoblotted for pY1068 (pEGFR), EGFR, pAKT, AKT, and β-Actin (loading control). **B.** The overall treatment plan for the transgenic mice with Doxycycline induced expression of human EGFR with a primary mutation (L858R) and secondary resistance mutation (T790M) (Tet-op-hEGFR-L858R-T790M). **C.** Graph shows percentage of change in tumor volume between vehicle (n = 6), Gefitnib (n=4), Hydroxychloroquine (HCQ) (n=6), and combination of Gefitnib and HCQ (n = 5) (left). Graph showing percentage of change in tumor volume between vehicle (n = 6), Paclitaxel (n=4), Hydroxychloroquine (HCQ) (n=6) and combination of Paclitaxel and HCQ (n = 6) (right). Significance in differences in tumor volumes with respect to vehicle treatment was determined by t‐test. Error bars denote SEM. ** and *** represent *p*-values <0.01 and <0.001, respectively. **D.** Immunohistochemistry analysis of Ki67 using tumors from above mentioned experiment for determination of cell proliferation in tumors. **E.** Graph showing the percentage of Ki67 staining with respect to the total nuclei from the immunohistochemical analysis of Ki67 from the panel D. Error bars denote S.D, ** represent *p*-value <0.01. Scale bar: 25 mm.

We next tested whether the combination of lysosomal inhibitors along with either TKIs or the microtubule stabilizing agent paclitaxel could overcome the oncogenicity of EGFR-TKI resistant cells such as H1975. We treated H1975 cells with vehicle, erlotinib (1 μM), paclitaxel (200 nM), and hydroxylchloroquine (10 μM), a compound structurally related to chloroquine, which is well tolerated in humans 26, and combinations of hydroxychloroquine with either erlotinib or paclitaxel for 16 hours, and checked for the activity of EGFR and downstream components such as AKT. Quite excitingly, we found that the combination of either erlotinib or paclitaxel with hydroxychloroquine showed decreased activation of EGFR compared to treatment with erlotinib or paclitaxel or hydroxychloroquine, as assessed by western blotting of pY1068 EGFR and the activity of AKT (Figure 6A).

To further test this observation in *in vivo* studies, we used a mouse model of doxycycline-inducible mutant EGFR (L858R) containing the T790M mutation. We used hydroxychloroquine 26, Gefitinib (EGFR TKI), and Paclitaxel (a microtubule stabilizing agent and chemotherapy drug), as single agents or in combination. We have used Gefitinib for our animal studies, but the mechanisms of action for both Gefitinib and Erlotnib are similar, reversibly binding to the ATP-binding sites of the EGFR kinase domain and halting EGFR-induced downstream signaling events 27, and also have similar efficacy in human patients 28. After five to six weeks of doxycycline treatment, upon confirmation of tumor growth in the lungs using MRI, mice were treated with vehicle, Paclitaxel (P), Gefitinib (G), Hydroxychloroquine (H), and combinations of Paclitaxel with Hydroxychloroquine (PH) or Gefitinib with Hydroxychloroquine (GH) (Figure 6B). Interestingly, similar to the data in the H1975 cell line, treatment with single agents such as Paclitaxel, Gefitinib, or Hydroxychloroquine alone could not reduce tumor size, but treatment with Hydroxychloroquine sensitized the tumors to better respond to Gefitinib or Paclitaxel, resulting in significantly reduced tumor size (Figure 6C, left and right graphs, respectively). Accordingly, we found that Ki67 staining (a marker for cellular proliferation) is significantly reduced upon combination treatment of Hydroxychloroquine with either Gefitinib or Paclitaxel, as compared to vehicle (*p*-values 0.0016 and 0.0030, respectively) or single-agent treatment with Gefitnib, Hydroxychloroquine, or Paclitaxel (Figure 6D and E). Taken together, our data show that the cells those are resistant for TKIs may be sensitized by the combination of TKIs with the microtubule stabilizing agent Paclitaxel.

## Discussion

In this study, we demonstrate that EGFR mutation in NSCLC results in a disorganized microtubule network, mislocalized endosomal pathway components, and resultant defective activated-receptor degradation. As a consequence, there are prolonged and excessive downstream pro-survival signals of the AKT and MAPK pathways. The defective microtubule network may result in global impaired processing of other proteins and drugs in EGFR mutants. This represents a novel perspective in oncogene-addicted cancers and identifies the tubulin/lysosomal pathway as a therapeutic target. Our observations correlate with clinical observations of a more benign clinical course, and increased sensitivity to treatment (chemotherapy and EGFR TKIs) 29 in EGFR mutation positive patients 30. Growth factors bind to specific receptors at the plasma membrane, internalize through receptor-mediated endocytosis, and are delivered to lysosomes for degradation. This entire migration of endocytic vesicles from plasma membrane to lysosomes takes place along microtubules. Microtubules radiate from the centrosomes, the primary MTOCs, and also organize other microtubule associated organelles such as Golgi and endoplasmic reticulum 31. Transport of endocytic vesicles and the perinuclear clustering of lysosomes at MTOC require an intact microtubule network 32-34, and depolymerization of microtubules induces impedance to receptor-mediated endocytosis 35-38. Microtubule integrity is an absolute requirement for the delivery of ligand-receptor cargo into the lysosomes 39. Microtubules are formed based on the assembly of α/β‑tubulin heterodimers. Microtubules are kept in equilibrium between free tubulin and the assembled state; paclitaxel binding to β-tubulin in the polymerized form results in stable microtubules 40. In our study, upon EGF treatment, we have shown that microtubules start to organise as early as 5 minutes in EGFR -WT cells but not in EGFR mutant (Figure 3C). We hypothesize that the early signaling events of EGF-EGFR-WT axis, such as early phosphorylation of microtubule associated proteins may be a key signal for microtubule organization which may be lacking in EGFR mutant cells. Further studies on the early phosphorylation events in EGFR may elucidate the signaling factors responsible for microtubule organization.

Importantly, the above findings may be extrapolated to other receptor tyrosine kinase mutated cancers. The sites of sensitizing mutations of various tyrosine kinases reveal a highly conserved affected site 41-44, located in the juxta-membrane regions which engage downstream adaptor proteins (shc and Grb2) or c-Cbl binding sites directing intracellular trafficking and degradation 20, 45-49. We demonstrated similar disorganization of expression of endosomal-lysosomal proteins in these oncogene cancers (Figure 4), implying that these cancers may also have defective trafficking, explaining their dependence and possibly shedding similar insights to mechanisms of acquired resistance to respective TKIs. Recent studies have also reported mutations in c-kit, MET, and PDGF receptors resulting in abnormal trafficking 50-53. However, there are multiple studies showing variety of other mechanisms of resistance to EGFR-TKIs and strategies to overcome TKI resistance 54-57.

This altered distribution of LAMP1 may serve as a useful surrogate for the presence of mutations across different cancer types. We demonstrated in a small cohort of patients (Table S1) that diffusely cytosolic LAMP1 was concordant with a sensitizing mutation, and therefore a predictor for response to EGFR tyrosine kinase inhibitors. This will need to be validated in a larger cohort.

From a therapeutic perspective, we demonstrated that lysosomal inhibitors disrupt lysosomal function and in doing so allow resistant cells to be sensitized to EGFR TKI therapy in cell culture and mice. We also show that lysosomal inhibitors in combination with microtubule stabilizing agents inhibit the growth of EGFR TKI resistant tumors in mice. To our knowledge, this is the first study to report the microtubule disorganization in any RTK mutant cancers and more interestingly, the efficacy of the combination of microtubule stabilizing agent and lysosome inhibitors delaying tumor progression. We also have shown that multiple subtypes of EGFR mutations confer disrupted microtubules in NSCLC, and taxol treatment could enhance the microtubule stability. This may increase either lysosomal delivery of EGFR mutants and/or sensitize EGFR TKI resistant mutants to TKIs. Further studies on the mechanism of microtubule targeting and TKI sensitivity should elucidate a pathway that may have a wider impact on the multiple cancers caused by awry RTKs signaling.

Interestingly, chloroquine has been shown to preferentially impede Myc-induced lymphoma in human Burkitt lymphoma using a transgenic mouse model, suggesting the benefits of using chloroquine in other oncogene addicted cancers 58. Also, Geser et. al had reported that there is a decreased rate of lymphoma in an African population that received chloroquine for malaria prophylaxis 59. The association between chloroquine use and reduced lymphoma development was strengthened further when the incidence of lymphoma was observed to increase two years after the malaria prophylaxis trial was completed. What was originally thought to be an anti-malarial effect of chloroquine is now believed to be mediated via its anti-cancer effect.

There are two small case series from a single center that has reported encouraging results on the use of chloroquine in combination with antineoplastic agents in patients with glioblastoma multiforme 60, 61. Furthermore, we found an increasing number of clinical trials registered at the National Cancer Institute website studying the efficacy of the combination of lysosomal inhibitors (chloroquine and hydroxychloroquine) and conventional chemotherapy. The lysosomal pathway may therefore represent a novel therapeutic target. Its efficacy in combination with micro-tubule agents for the treatment of cancers should be studied.

The discovery of oncogene addicted cancers has revolutionized care in cancer. Our findings provide a novel and complementary perspective to the understanding of tumors with mutated tyrosine kinases. Importantly, this insight opens up the possibility of pharmacological intervention of microtubule organization and the lysosomal pathway as a novel therapeutic option for these cancers.

## Supplementary Material

Supplementary figures and table.
