# Supplementary material for: Targeting microtubules sensitizes drug resistant lung cancer cells to lysosomal pathway inhibitors
Source: Theranostics. 2020 Feb 3;10(6):2727–43. doi: 10.7150/thno.38729 (PMC7052910; doi:10.7150/thno.38729)
Supplement: Supplementary file 1 — Supplementary figures and table. [file thnov10p2727s1.pdf]

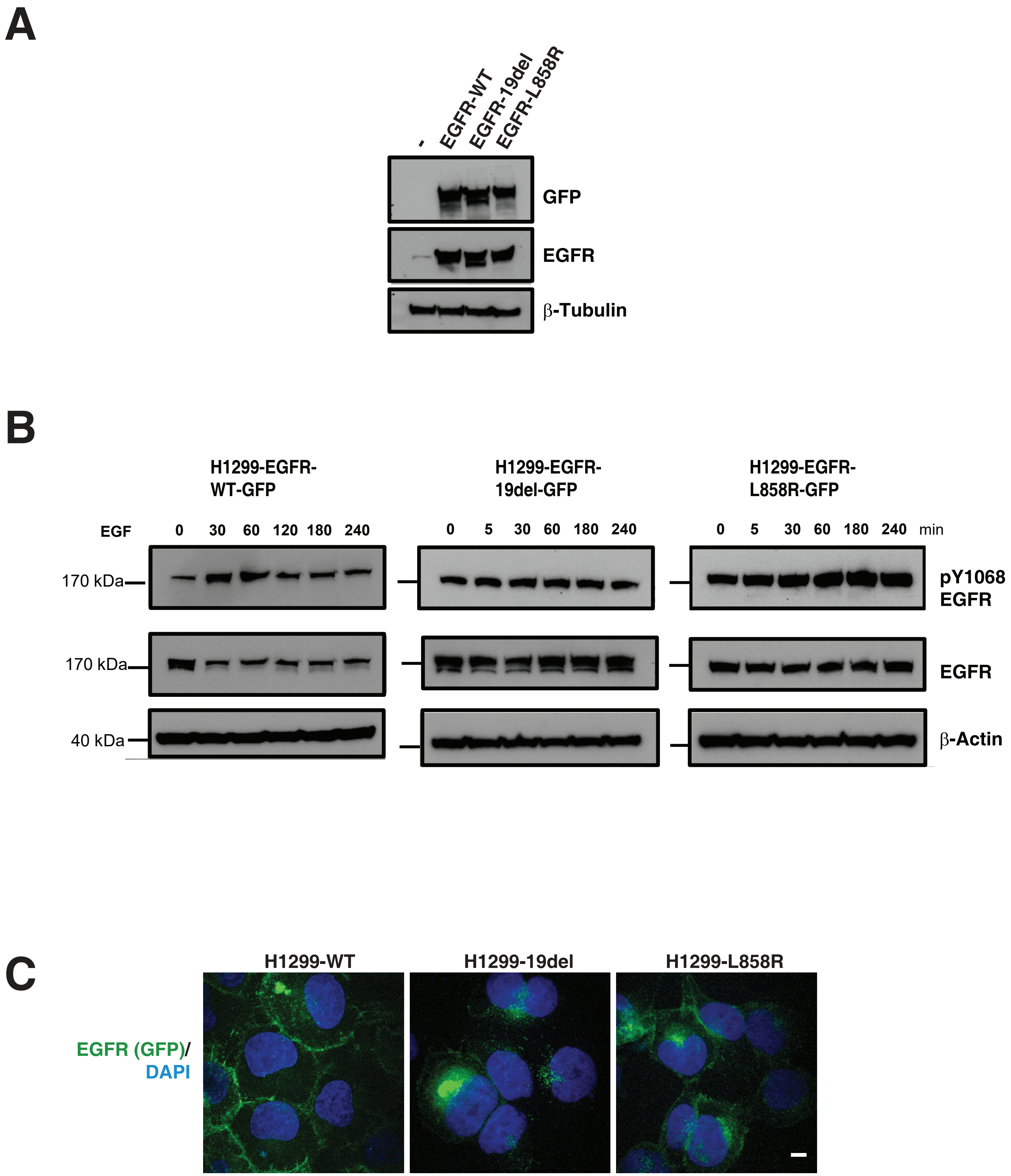

A
-
EGFR-WT
EGFR-19del
EGFR-L858R
GFP
EGFR
β-Tubulin
B
H1299-EGFR-WT-GFP
H1299-EGFR-19del-GFP
H1299-EGFR-L858R-GFP
EGF
0 30 60 120 180 240
0 5 30 60 180 240
0 5 30 60 180 240 min
170 kDa
170 kDa
40 kDa
pY1068 EGFR
EGFR
β-Actin
C
H1299-WT
H1299-19del
H1299-L858R
EGFR (GFP)/
DAPI

Figure S1: Validation of H1299 cells stably transfected with EGFR. **A,** Immunoblot analysis of GFP and total EGFR in stably transfected cells expressing EGFR WT, exon 19 deletion, and L858R mutation tagged with a C-terminal GFP, respectively. β–tubulin served as a loading control. **B,** EGFR degradation in stable cell lines: H1299 stable cells expressing EGFR-WT, EGFR-19del, and EGFR-L858R were serum starved with for 12-16 hours, treated with EGF (100 ng/ml), and harvested at various time points mentioned. Cell lysates collected were immunoblotted for pY1068-EGFR, total EGFR, and β-actin (loading control). **C,** EGFR WT, as well as 19-del- and L858R mutation- containing stably transfected cells were fixed with methanol and recorded using confocal microscopy. DAPI stains the nucleus. Scale bar: 10 μm.

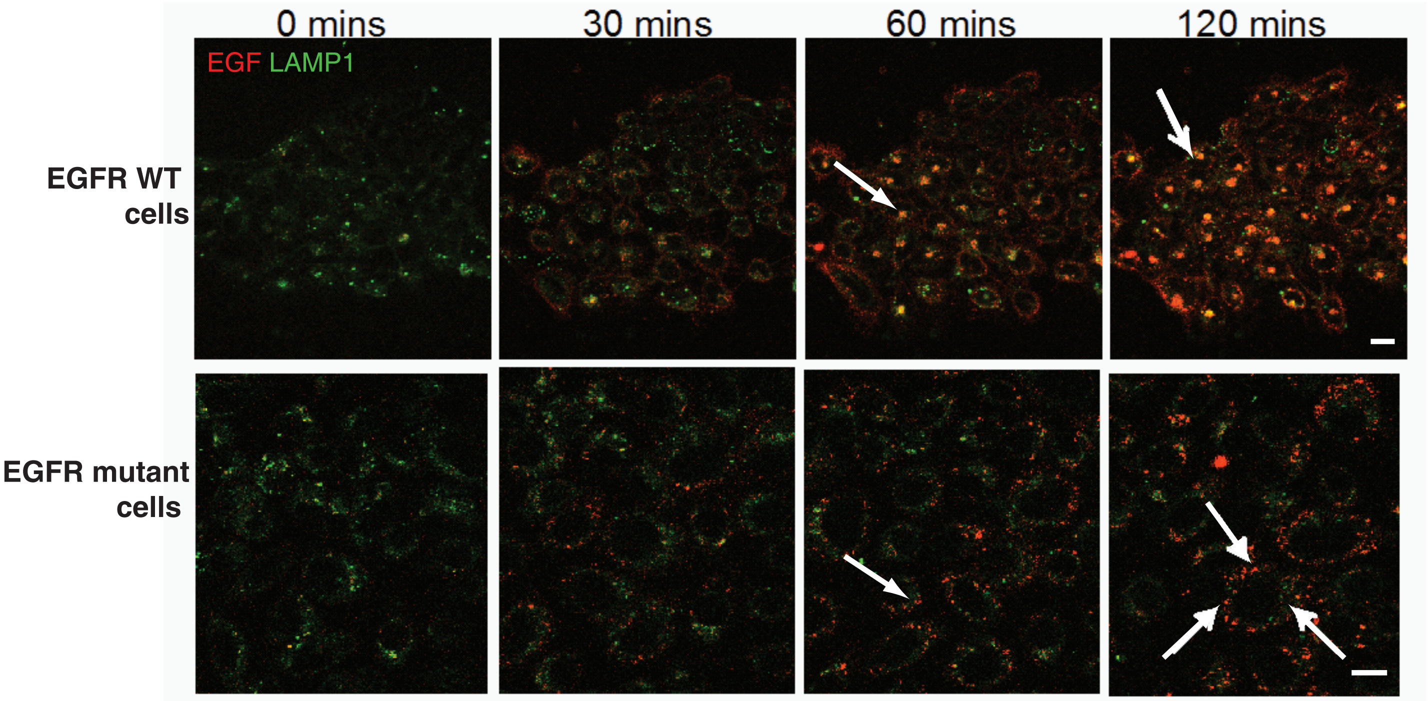

0 mins
30 mins
60 mins
120 mins
EGF LAMP1
EGFR WT cells
EGFR mutant cells

Figure S2: Mutation status rather than EGFR levels cause delay in EGF maturation to lysosomes. EGFR WT overexpressing cells (A431) and EGFR mutant cells (PC9) were serum starved overnight, treated with texas-red conjugated EGF, and fixed using paraformaldehyde at 0, 30, 60, and 120 minutes. Immunofluorescence of LAMP1 (green) was performed on the fixed cells. The white arrows in the top row indicate the colocalization of EGF with LAMP1, and in the bottom row indicate no-colocalization at 60 and 120 minutes. Scale bar: 10 μm.

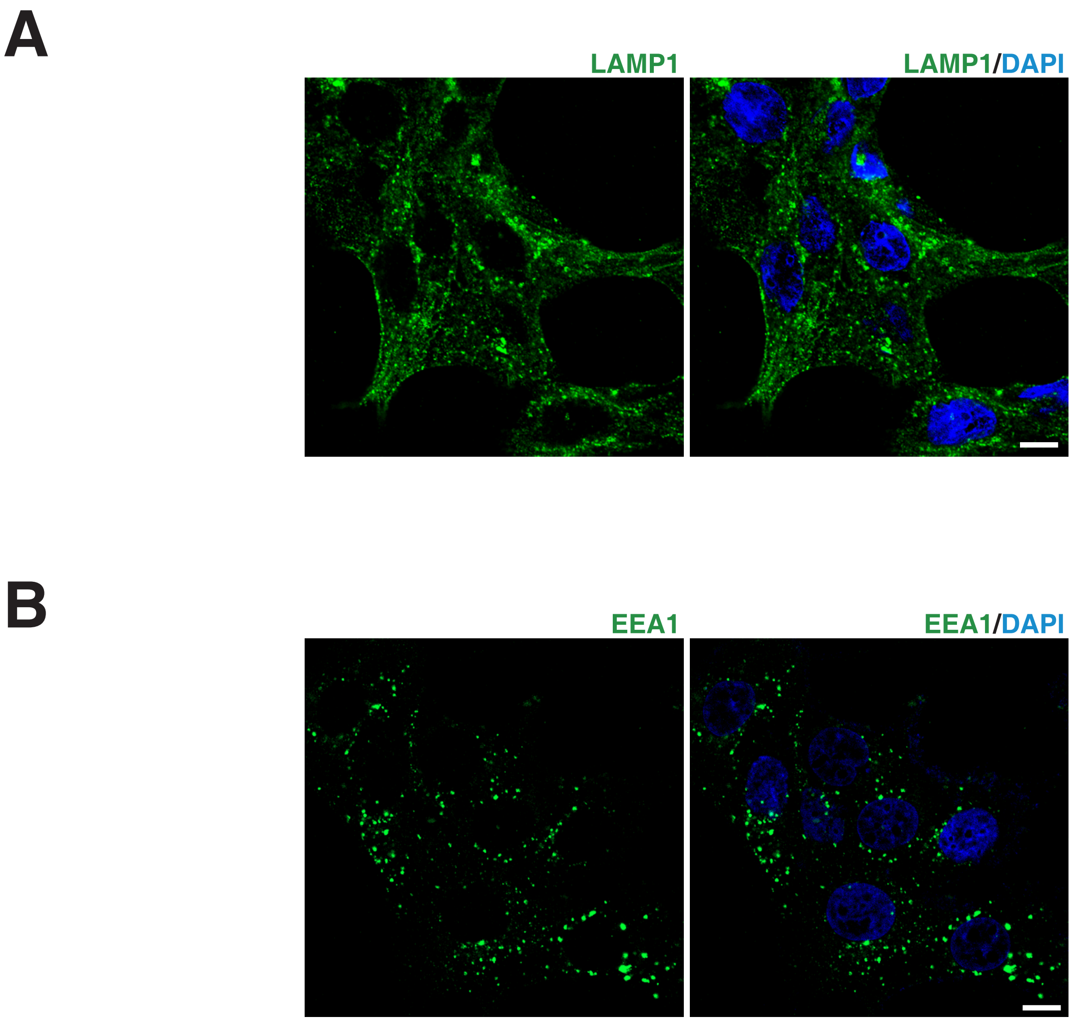

A
LAMP1
LAMP1/DAPI
B
EEA1
EEA1/DAPI

Figure S3: Distribution of lysosomes and early endosomes in H1975 cells.

Immunofluorescence staining of (**A**) LAMP1 (lysosomes) and (**B**) EEA1 (early endosomes) in H1975 cells, a TKI resistant NSCLC cell line that harbors L858R and T790M mutations. DAPI stain represents the nucleus. Scale bar: 10 µm.

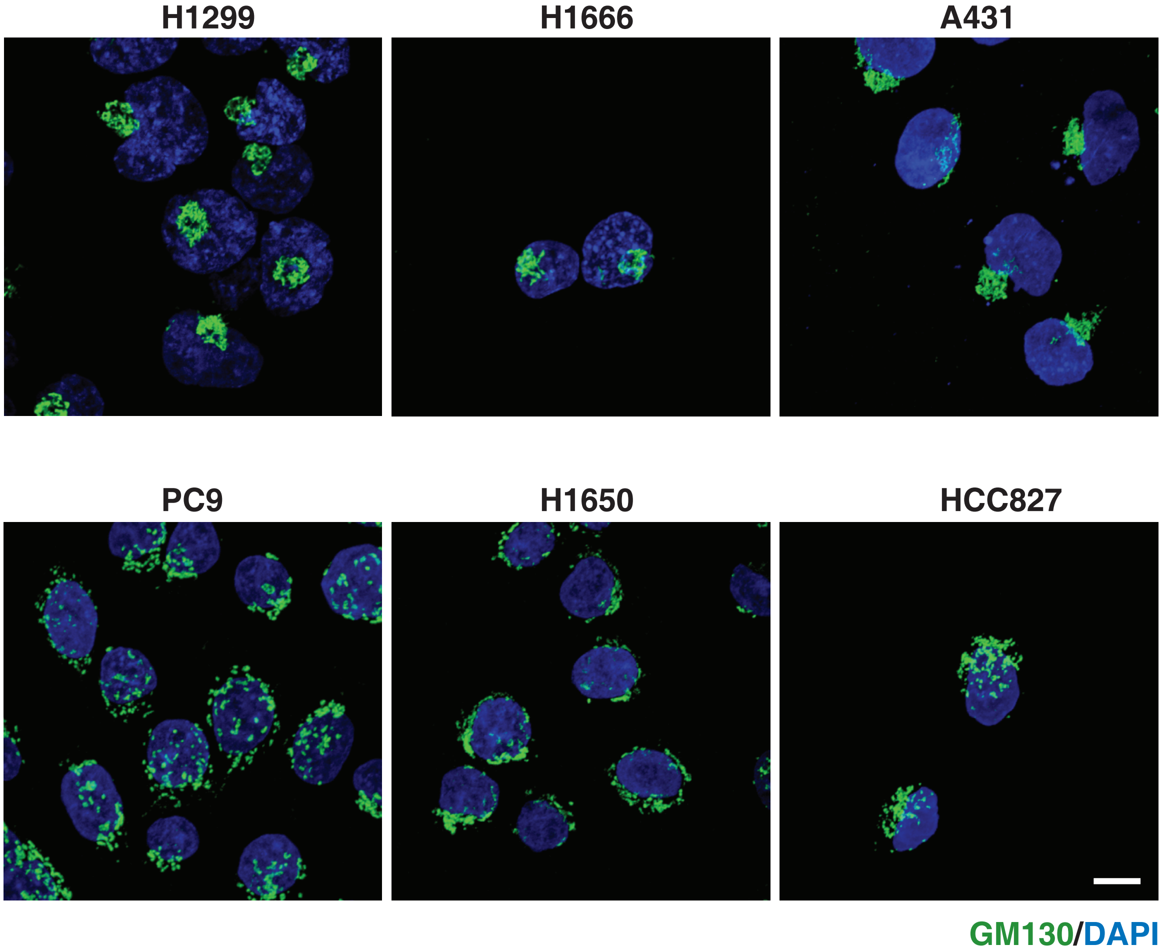
H1299
H1666
A431
PC9
H1650
HCC827
GM130/DAPI

Figure S4: EGFR mutation causes fragmentation of Golgi network. Immunofluorescence staining of GM130 protein (a cis-Golgi matrix protein) in NSCLC and A431 cells. NSCLC cells with EGFR WT (H1299 and H1666) and EGFR mutant (PC9, H1650, and HCC827) were used. A431 cells represent overexpression of EGFR WT. DAPI stain represents nucleus. Scale bar: 10 µm.

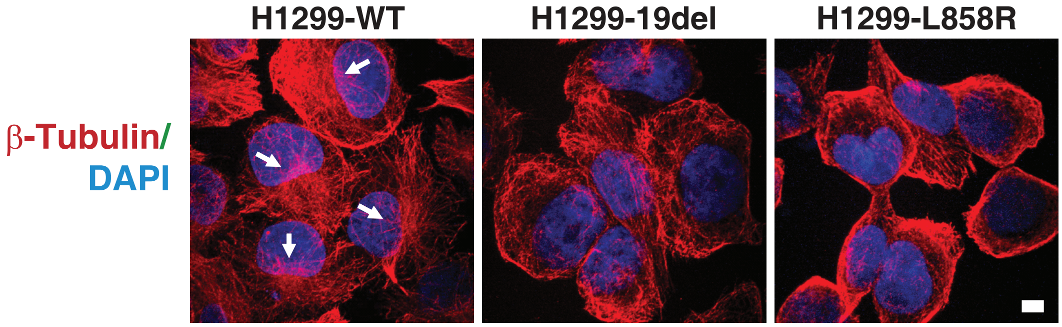
H1299-WT
H1299-19del
H1299-L858R
β-Tubulin/
DAPI

Figure S5: EGFR mutants cause disruption of microtubule network in H1299 stable cells. EGFR WT, as well as 19-del- and L858R mutation- containing stably transfected cell lines (from supplementary figure 1C) were used for immunofluorescence of β−Tubulin (red). The white arrows point to the microtubule organizing centre (MTOC). DAPI stains the nucleus. Scale bar: 10 μm.

Table S1

Microscopic appearance of LAMP1 in tumors samples of patients with sensitizing EGFR mutations and their response to EGFR tyrosine kinase inhibitors (Gefitinib)

**Patient ID**	**Mutation type**	**Microscopic appearance of LAMP1**	**Response to EGFR TKI**	**Duration of response**
CKL	Ex 19 del 746-750	Diffusely cytoplasmic	PR	11 months (+)
SLW	Ex 19 del 746-750	Diffusely cytoplasmic	PR	12 months
YGW	Ex 19 del 746-750	Diffusely cytoplasmic	PR	24 months (+)
LC	Ex 21 L858R	Diffusely cytoplasmic	PR	12 months
YCE	Ex 21 L858R	Diffusely cytoplasmic	SD	4 months
LAB	Ex 21 L858R	Diffusely cytoplasmic	SD	10 months
SS	Ex 19 del 746-751 insA	Diffusely cytoplasmic (Weak)	SD	10 months
TMC	L858R; V738F	Diffusely cytoplasmic (Weak)	SD	6 months
NSS	Wild-type	Focal	PD	-
KBM	Wild-type	Focal	-	-

PR- Partial response; SD- Stable disease; PD- Progressive disease.

+ denotes ongoing treatment and response
